# Singlet Oxygen-Mediated Degradation of 17β-Estradiol by Peracetic Acid Activated over Co Species Confined in Carbon Nanotubes

**DOI:** 10.3390/molecules31172946

**Published:** 2026-08-22

**Authors:** Jie Teng, Yuzhen Zhang, Xiangbo Ma, Wencheng Zhu, Pu Li, Mingguo Peng

**Affiliations:** School of Urban Construction, Changzhou University, Changzhou 213164, China; tammytengjie@cczu.edu.cn (J.T.); zhangyuzhen4683@163.com (Y.Z.); mmmaxiangbo@163.com (X.M.); 18921526303@163.com (W.Z.)

**Keywords:** 17β-estradiol, peracetic acid activation, nanoconfinement, cobalt-based catalyst, singlet oxygen

## Abstract

17β-Estradiol (E2), a highly bioactive steroid estrogen, poses potential ecological risks even at trace concentrations in aquatic environments. In this study, a nanoconfined Co-based catalyst (Co@C_in_) was employed to activate peracetic acid (PAA) for efficient E2 degradation. Microscopic and spectroscopic characterizations indicated that Co-containing species were highly dispersed within the CNT-based architecture, while the graphitic tubular framework was largely preserved. Compared with PAA alone, CNTs, Co@C_in_ alone, and the externally loaded Co@C_out_/PAA system, Co@C_in_/PAA exhibited substantially faster E2 degradation. Pseudo-first-order kinetic analysis further demonstrated the enhanced degradation kinetics of the internally confined system. Importantly, ICP analysis showed comparable Co loadings for Co@C_in_ and Co@C_out_, while Co@C_in_ retained a markedly higher Co-normalized apparent kinetic activity, indicating that its enhanced performance cannot be explained simply by differences in total Co loading. Moreover, Co@C_in_ exhibited substantially lower Co leaching than Co@C_out_ and maintained considerable catalytic activity over five consecutive cycles, demonstrating improved stability of the confined Co species. The degradation performance was influenced by initial E2 concentration, catalyst dosage, PAA concentration, and pH, while Cl^−^ and NO_3_^−^ showed negligible effects and humic acid and CO_3_^2−^ caused only moderate inhibition. Scavenging experiments and controlled TEMP-EPR measurements with appropriate blank and control systems supported a dominant contribution of singlet oxygen (^1^O_2_), with radical pathways playing only minor roles. The enhanced performance is therefore associated with the nanoconfined reaction environment, which promotes efficient PAA activation while stabilizing the Co species. This work extends nanoconfinement-regulated PAA oxidation to the treatment of the highly bioactive steroid estrogen E2 and provides a quantitative assessment of the activity–stability advantages of internally confined Co species.

## 1. Introduction

17β-estradiol (E2) is a representative natural steroid estrogen and has been widely recognized as an emerging contaminant of concern in aquatic environments. It is mainly released from human and animal excretion, hospital wastewater, aquaculture effluents, and municipal wastewater treatment plant discharges, allowing its continuous input into receiving water bodies [1,2,3,4]. Unlike many conventional organic pollutants, the ecological risk of E2 is not solely determined by its environmental concentration, but is strongly associated with its high biological activity and endocrine-disrupting potency [5,6]. Previous studies have shown that steroid estrogens, even at ng/L–μg/L levels, can induce feminization of male fish, reproductive disorders, impaired gonadal development, and population-level reproductive decline in aquatic organisms [7,8,9,10]. Therefore, developing efficient treatment technologies capable of destroying the steroid estrogen structure and reducing the endocrine-disrupting risk of E2 is of great significance for controlling pollutants characterized by low-dose effects, high bioactivity, and chronic exposure.

Various technologies have been developed for E2 removal, including adsorption, biodegradation, membrane separation, and advanced oxidation processes (AOPs) [11,12,13,14,15]. Among them, AOPs have attracted increasing attention because they can generate highly reactive species in situ to attack and transform the molecular structure of E2 [16,17,18,19,20,21]. Peracetic acid (PAA), an environmentally friendly oxidant with both oxidation and disinfection capability, has recently emerged as a promising oxidant for water and wastewater treatment. Compared with conventional oxidants such as H_2_O_2_, peroxymonosulfate (PMS), and peroxydisulfate (PDS), PAA offers several advantages, including a relatively high redox potential, lower O–O bond dissociation energy, reduced risk of halogenated disinfection by-product formation, and simultaneous disinfection benefits [22,23,24,25,26,27]. With the growing understanding of PAA activation chemistry, nonradical oxidation pathways have received increasing attention. Recent studies have demonstrated that high-valent Co(IV)-oxo species, interfacial electron transfer, and singlet oxygen (^1^O_2_) may participate in PAA activation and contaminant oxidation [28,29,30]. These findings suggest that Co-based materials are not only efficient PAA activators, but can also regulate the activation pathway of PAA through modulation of the electronic structure of active sites and the interfacial reaction microenvironment [31,32,33]. Thus, an important challenge in Co/PAA systems is not merely to maximize reactive-species generation, but to regulate the dominant reaction pathway and improve oxidant utilization through rational catalyst and interfacial design [34,35].

The molecular structure of E2 contains an electron-rich phenolic A-ring and a secondary hydroxyl group at C17, both of which may serve as susceptible sites during oxidative transformation. Previous studies have reported hydroxylation, oxidation of the C17 hydroxyl group to ketone-containing products such as estrone, further oxidation of phenolic intermediates, and eventual aromatic-ring cleavage during E2 oxidation. Therefore, identifying the dominant reactive species provides a useful basis for discussing the possible molecular transformation behavior of E2, although direct identification of transformation products is still required for definitive pathway assignment.

In heterogeneous Fenton-like reactions, reactive species often suffer from short lifetimes and limited diffusion distances, and they can be readily consumed by background water matrices before effectively reacting with target pollutants [36,37,38,39]. Nanoconfinement provides a promising strategy to address this limitation. By spatially confining catalytic active sites, oxidants, and pollutants within nanoscale domains, the migration distance between reactive species generation sites and target molecules can be shortened, thereby improving the utilization efficiency of short-lived species and altering interfacial reaction pathways [40]. Carbon nanotubes (CNTs), which feature hollow nanochannels, high specific surface area, favorable electron transport properties, and distinct electronic differences between their inner and outer surfaces, are ideal platforms for constructing nanoconfined reactors [41]. Previous studies have established that confining Co_3_O_4_ nanoparticles inside CNT nanochannels can markedly enhance PAA activation and contaminant oxidation by altering the local reaction environment and mass-transfer behavior. In these reported systems, nanoconfinement was further shown to shift PAA activation away from conventional radical-dominated pathways toward a predominantly ^1^O_2_-mediated nonradical oxidation process [42]. In such confined systems, CNT nanochannels can promote local enrichment of reactants and shorten the transport distance between reactive species and target molecules, while interactions between Co species and the inner CNT wall may alter the local electronic environment and facilitate PAA activation [43]. Nevertheless, although the general advantages of Co/CNT nanoconfinement for PAA activation have been demonstrated, its systematic application to the oxidation of highly bioactive steroid estrogens such as E2 remains insufficiently evaluated. In particular, a quantitative comparison between internally confined and externally loaded Co species in terms of E2 degradation kinetics, Co-normalized catalytic performance, and metal stability is still lacking.

Based on this rationale, the present study systematically evaluated a nanoconfined Co/CNT catalyst (Co@C_in_) for PAA activation and E2 oxidation, with an externally loaded Co@C_out_ catalyst employed as a structural reference. The E2 degradation performance of Co@C_in_/PAA was systematically evaluated through kinetic analysis, variation of reaction conditions, water-matrix experiments, and cycling tests, while the actual Co loadings, Co-normalized apparent activities, and Co leaching of Co@C_in_ and Co@C_out_ were further compared to distinguish confinement-related effects from differences in total Co content. Furthermore, radical/nonradical scavenging experiments and controlled TEMP-EPR measurements with appropriate blank and control systems were employed to evaluate the contribution of ^1^O_2_, while possible E2 transformation routes were discussed based on the experimentally supported oxidation chemistry and previously reported E2 transformation behavior.

## 2. Results

### 2.1. Characterization of Co@C_in_

**Figure 1:**
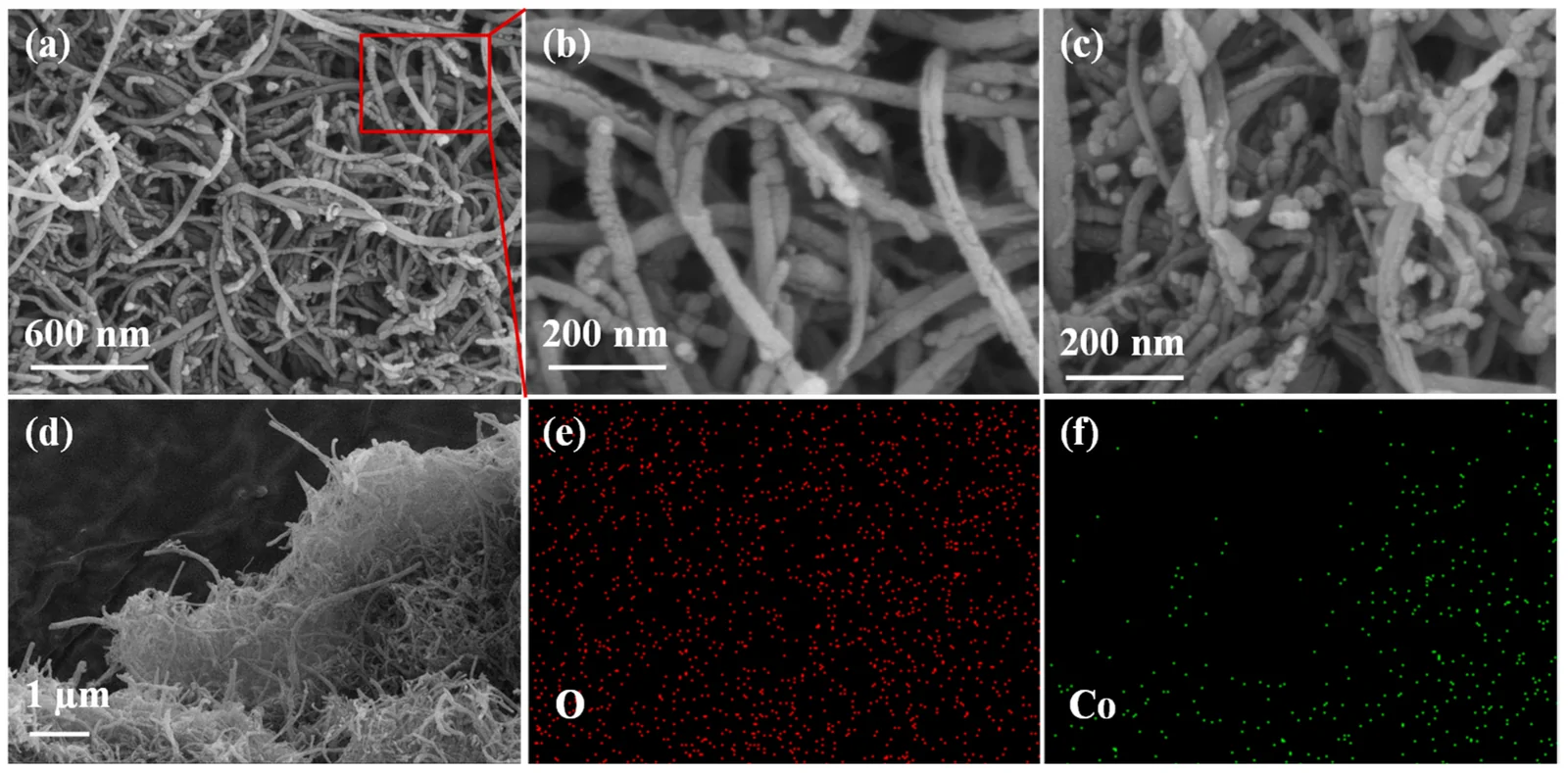
SEM images of Co@C_in_ at (**a**) 50,000× and (**b**) 100,000× magnifications; (**c**) SEM images of Co@C_out_ at 100,000× magnification; (**d**) SEM image of the selected region for elemental mapping; and (**e**,**f**) EDS elemental maps of O and Co for Co@C_in_.

The morphology and elemental distribution of the prepared catalysts were examined by SEM with EDS. As shown in Figure 1a,b Co@C_in_ retained the typical three-dimensional entangled network of CNTs. The interconnected tubular architecture was well preserved after acid treatment and Co loading, without obvious collapse or severe aggregation, indicating that the synthetic process did not destroy the one-dimensional carbon framework. At higher magnification, the external surface of Co@C_in_ appeared relatively clean and smooth, with no apparent accumulation of large Co-containing particles on the CNTs walls (Figure 1b). This observation suggests that Co species were not predominantly deposited on the outer surface of CNTs, but were more likely introduced in a highly dispersed or spatially confined manner. In contrast, Co@C_out_ displayed numerous particle-like deposits attached to the external surface of CNTs (Figure 1c), implying that, without sufficient opening of the nanotube channels, the Co precursor preferentially nucleated and grew on the outer walls, leading to an externally loaded configuration. The EDS mapping images of Co@C_in_ reveal broadly distributed O and Co signals throughout the selected region (Figure 1d–f), supporting the incorporation and dispersion of Co-containing species within the CNT-based matrix without obvious large-scale aggregation.

**Figure 2:**
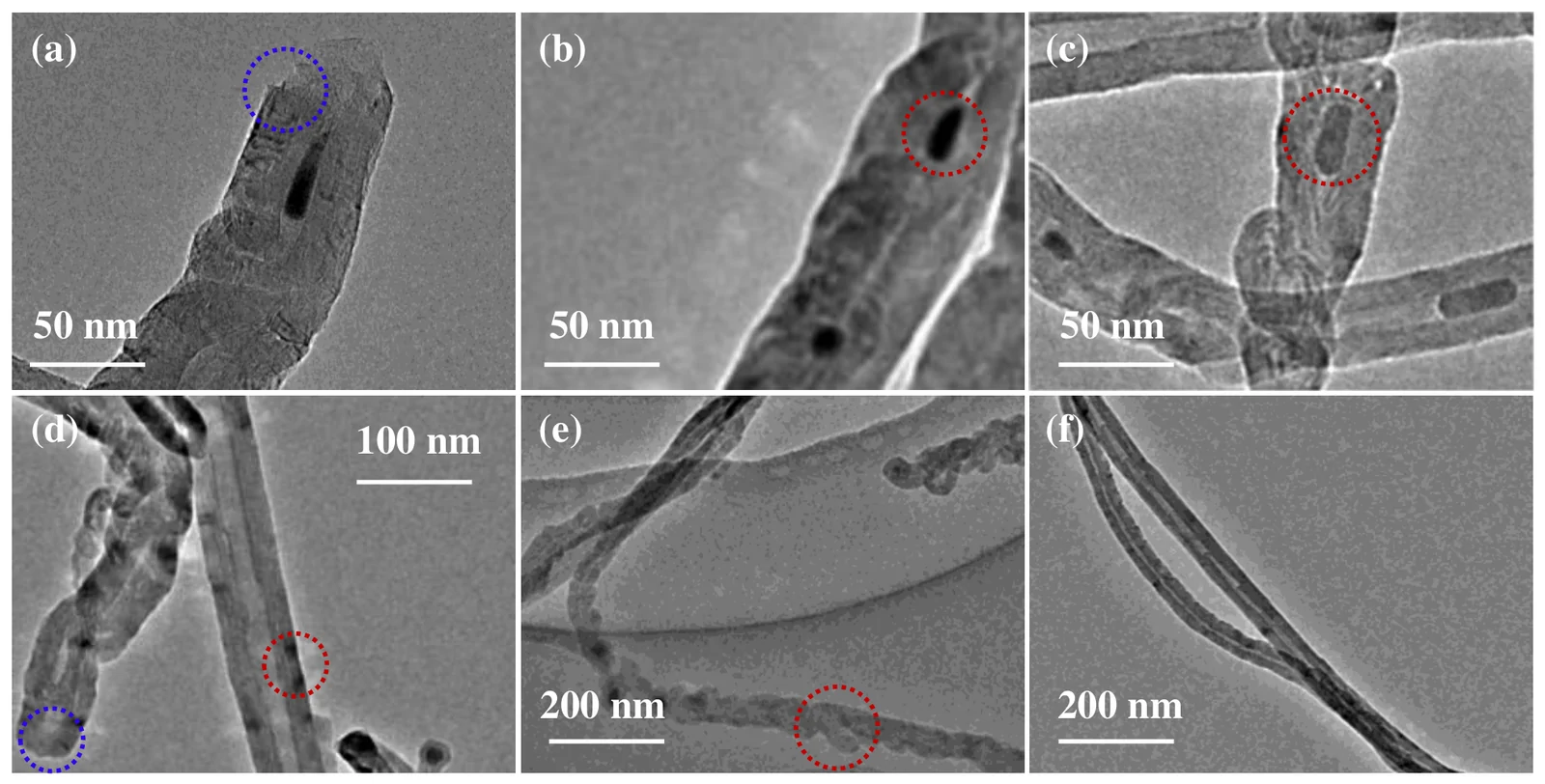
TEM images of the (**a**–**c**) Co@C_in_, (**d**,**e**) Co@C_out_, and (**f**) pristine CNTs. The blue circles in panels (**a**,**d**) indicate representative opened and closed CNT ends, respectively, while the red dashed circles highlight representative Co-containing domains located within or close to the internal CNT channels.

TEM observations were conducted to further clarify the spatial location of Co species within the CNTs architecture. As shown in Figure 2a–c, Co@C_in_ exhibited a hollow tubular structure with clearly opened nanotube ends, and several dark contrast domains were observed inside the CNT nanochannels or near the inner cavity. For Co@C_in_, several dark-contrast domains were observed along the CNT channels and within the projected inner-cavity regions, suggesting that a considerable fraction of the Co-containing species was associated with the confined CNT structure. In comparison, Co@C_out_ exhibited more readily visible particles associated with the outer CNT walls. Although conventional TEM provides useful morphological evidence for the different spatial distributions of Co species, the two-dimensional projection nature of TEM limits an unambiguous determination of nanoparticle locality based solely on these images. The red dashed circles highlight representative Co species located within or close to the internal channels, suggesting that the acid-assisted opening of CNTs facilitated precursor diffusion and subsequent confined growth during thermal treatment. By contrast, Co@C_out_ showed more obvious dark particles attached to the outer walls of CNTs (Figure 2d,e), indicating that the Co precursor mainly nucleated on the external surface when the nanotube channels were not sufficiently opened. This externally exposed configuration is distinct from the nanoconfined structure of Co@C_in_. Pristine CNTs displayed smooth hollow tubes without visible Co-containing particles (Figure 2f), further confirming that the dark domains observed in Co@C_in_ and Co@C_out_ originated from the introduced Co species. Overall, the TEM observations, together with the SEM-EDS analysis, support distinct spatial distributions of Co-containing species in Co@C_in_ and Co@C_out_ and are consistent with a predominantly confined configuration for Co@C_in_.

**Figure 3:**
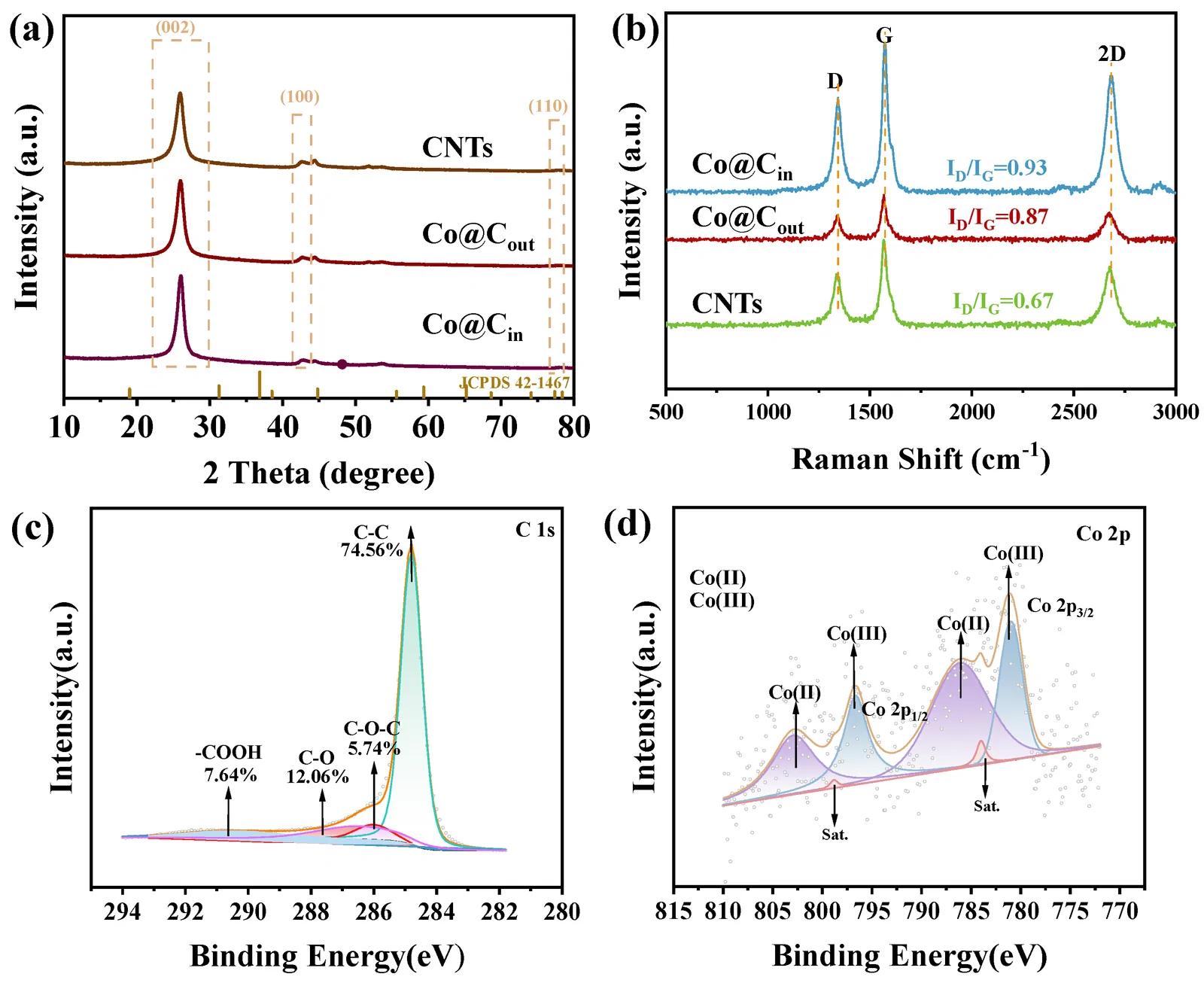
(**a**) XRD patterns and (**b**) Raman spectra of Co@C_in_, Co@C_out_, and CNTs; high-resolution XPS spectra of Co@C_in_: (**c**) the C1s peak and (**d**) the Co 2p peak.

XRD, Raman, and XPS analyses were further conducted to elucidate the crystalline structure, defect characteristics, and surface chemical states of the catalysts. As shown in Figure 3a, pristine CNTs exhibited two characteristic diffraction peaks at approximately 26° and 43°, which are assigned to the (002) and (100) planes of graphitic carbon, respectively. After the introduction of Co species, the characteristic diffraction features of the CNT framework were still retained in both Co@C_in_ and Co@C_out_. However, no distinct diffraction peaks attributable to highly crystalline cobalt oxide phases were clearly resolved, particularly for Co@C_in_. This observation suggests that the Co-containing species are likely present as small, highly dispersed, and relatively low-crystallinity domains. For Co@C_in_, nanoconfinement within the CNT channels may further restrict the growth and crystallization of the Co-containing domains, resulting in weak and broadened diffraction signals that are difficult to distinguish from the CNT background. Therefore, the XRD results alone are insufficient for unambiguous identification of the crystalline phase and spatial location of the confined Co species. Their presence and chemical states are further supported by microscopic observations and XPS analysis. The structural evolution of the CNT framework was further investigated by Raman spectroscopy (Figure 3b). All samples exhibited the characteristic D and G bands at approximately 1350 and 1580 cm^−1^, respectively. The D band is generally associated with defects and structural disorder in sp^2^-carbon materials, whereas the G band originates predominantly from the in-plane vibration of sp^2^-bonded carbon atoms [44,45]. Accordingly, the intensity ratio of the D and G bands (I_D_/I_G_) was used as a comparative indicator of structural disorder in the CNT-based samples. The I_D_/I_G_ values were 0.67, 0.87, and 0.93 for pristine CNTs, Co@C_out_, and Co@C_in_, respectively. The increased I_D_/I_G_ values after Co incorporation indicate an increased degree of structural disorder in the CNT framework. The slightly higher I_D_/I_G_ value of Co@C_in_ than that of Co@C_out_ may be associated with the additional structural perturbation caused by CNT opening and confined Co loading. Similar changes in the Raman I_D_/I_G_ ratio have been reported for Co oxide species confined within CNT nanochannels [43]. XPS analysis was then performed to determine the surface composition and valence states of Co@C_in_. The high-resolution C1s spectrum (Figure 3c) could be deconvoluted into C–C/C=C, C–O, and –COOH species, confirming the introduction of oxygen-containing functional groups during acid treatment. These functionalities may strengthen the interaction between Co species and the carbon matrix. In the Co 2p spectrum (Figure 3d), the Co 2p^3/2^, Co 2p^1/2^, and corresponding satellite peaks were clearly observed. The coexistence of Co(II) and Co(III) indicates that Co species in Co@C_in_ mainly existed as mixed-valence cobalt oxides. Such Co(II)/Co(III) redox couples are beneficial for electron transfer and may play a key role in activating PAA for E2 degradation. To ensure a fair comparison between the internally and externally loaded Co catalysts, the actual Co contents of Co@C_in_ and Co@C_out_ were further quantified by ICP-OES. The Co contents were determined to be 24.3 and 23.8 wt%, respectively. The very similar Co loadings indicate that the two catalysts contained comparable total amounts of Co. Therefore, the pronounced difference in their catalytic performances cannot be simply attributed to a difference in the overall Co loading.

### 2.2. Catalytic Performance

**Figure 4:**
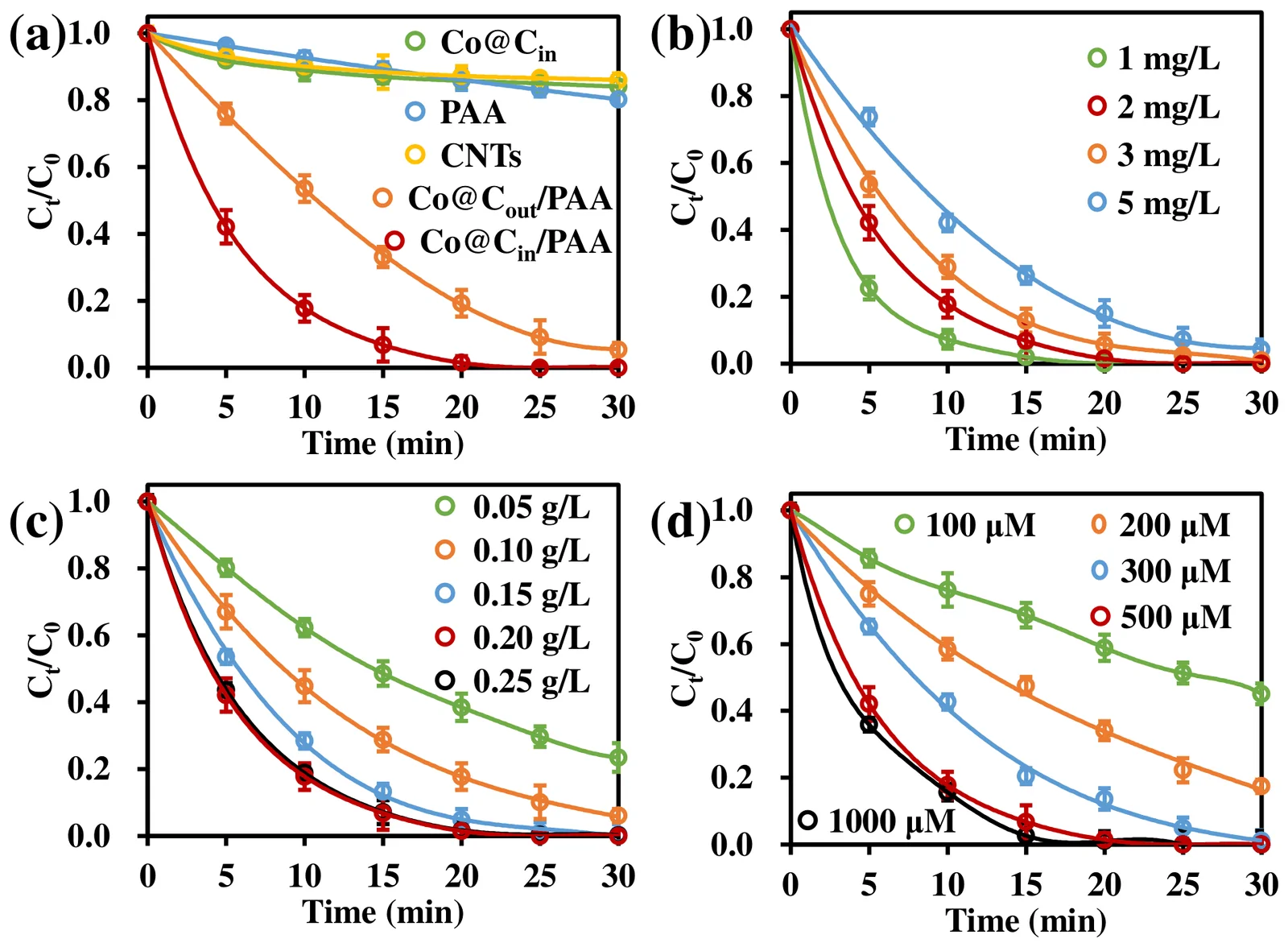
E2 degradation performance under different conditions: (**a**) different reaction systems, (**b**) initial E2 concentration, (**c**) Co@C_in_ dosages, and (**d**) PAA concentrations.

The catalytic activity of the prepared samples was evaluated using E2 as the target pollutant in the PAA-based oxidation system. As shown in Figure 4a, negligible E2 removal was observed in the presence of PAA alone, CNTs alone, or Co@C_in_ alone, indicating that direct PAA oxidation and physical adsorption contributed only marginally to E2 removal under the tested conditions. In contrast, the Co@C_in_/PAA system exhibited markedly enhanced degradation performance, achieving nearly complete E2 removal within a short reaction time. Co@C_out_/PAA also promoted E2 degradation, but its removal rate was obviously lower than that of Co@C_in_/PAA. This comparison highlights the positive role of the nanoconfined Co sites inside CNT channels, which may provide a more favorable microenvironment for PAA activation and interfacial electron transfer.

To quantitatively compare the catalytic performances of the two systems, the experimental data were further analyzed using a pseudo-first-order kinetic model. The apparent rate constant (k_obs_) of the Co@C_in_/PAA system was 0.204 min^−1^ (R^2^ = 0.984), which was approximately 2.48 times that of the Co@C_out_/PAA system (0.0823 min^−1^, R^2^ = 0.982). Correspondingly, the calculated half-life of E2 decreased from 8.43 min in the Co@C_out_/PAA system to 3.40 min in the Co@C_in_/PAA system, quantitatively confirming the substantially enhanced E2 degradation kinetics of Co@C_in_/PAA.

Notably, ICP-OES analysis revealed comparable Co contents of 24.3 wt% for Co@C_in_ and 23.8 wt% for Co@C_out_. To further minimize the influence of the slight difference in Co loading, the kinetic constants were normalized to the actual Co contents. At a catalyst dosage of 0.20 g L^−1^, the Co-normalized apparent rate constants were calculated to be 4.20 and 1.73 L g-Co^−1^ min^−1^ for Co@C_in_/PAA and Co@C_out/_PAA, respectively. Thus, Co@C_in_ retained an approximately 2.43-fold higher Co-normalized apparent kinetic activity than Co@C_out_. These results indicate that the superior catalytic performance of Co@C_in_ cannot be explained merely by differences in the total Co loading, but is strongly associated with the nanoconfined configuration and local reaction environment of the Co species.

The influence of key operational parameters was further investigated. As the initial E2 concentration increased from 1 to 5 mg/L, the degradation rate gradually decreased (Figure 4b), which can be attributed to the limited availability of reactive species and catalytic sites at higher pollutant loadings. Nevertheless, efficient E2 removal was still achieved within 30 min, demonstrating the strong oxidative capacity of the Co@C_in_/PAA system. Increasing the catalyst dosage from 0.05 to 0.20 g/L significantly accelerated E2 degradation (Figure 4c) owing to the increased number of accessible Co active sites. A further increase to 0.25 g/L produced only a slight improvement, suggesting that 0.20 g/L was sufficient for effective PAA activation. Similarly, E2 degradation was enhanced as the PAA concentration increased from 100 to 500 μM (Figure 4d), while excessive PAA provided limited additional benefit, probably because of the saturation of catalytic activation sites or self-quenching of reactive intermediates.

**Figure 5:**
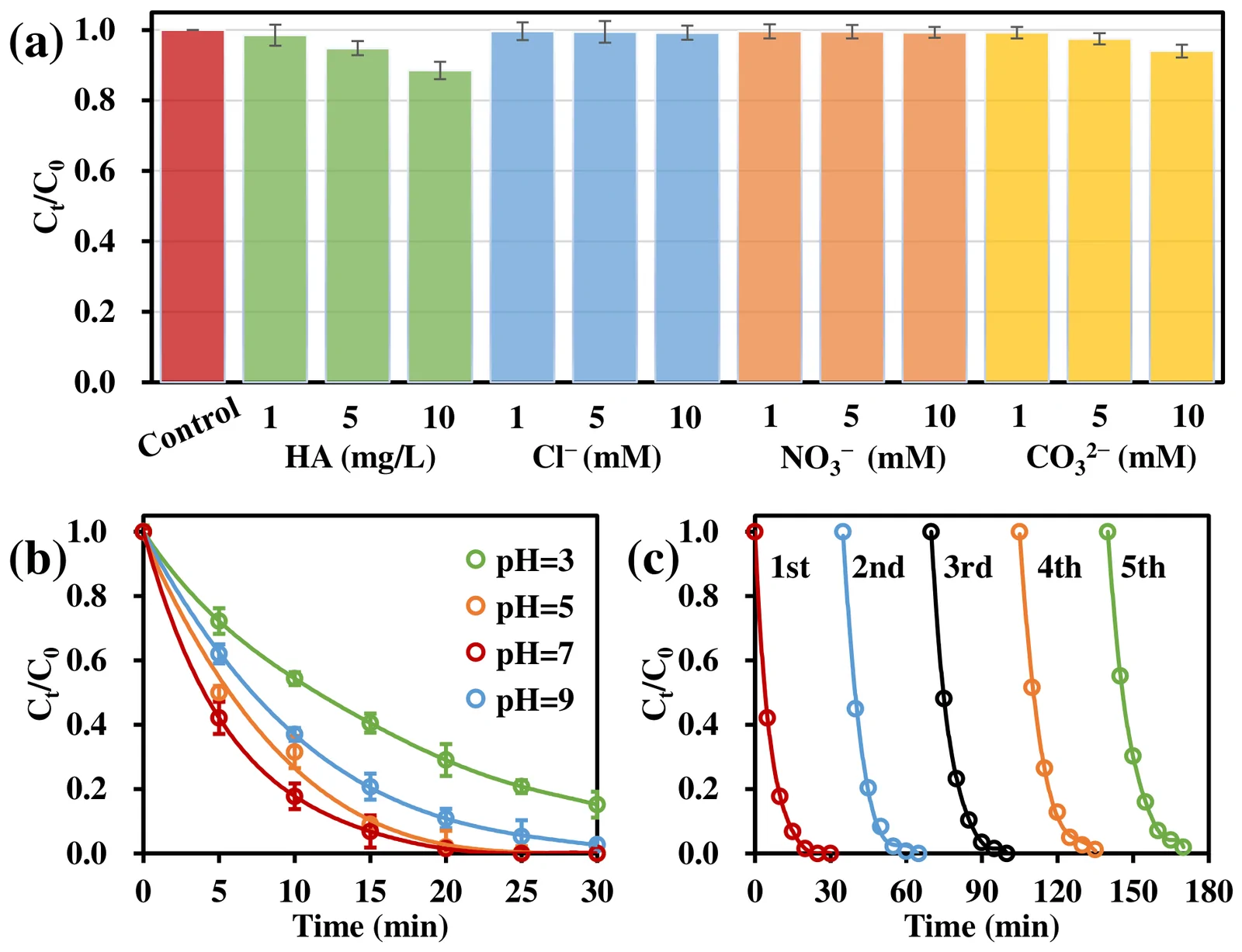
Effect of (**a**) water matrix components (HA, Cl^−^, NO_3_^−^, CO_3_^2−^) and (**b**) initial pH on E2 degradation; (**c**) cyclic performance test of Co@C_in_.

The effects of common water matrix components were also examined to assess the environmental applicability of the system. As shown in Figure 5a, Cl^−^ and NO_3_^−^ had negligible influences on E2 degradation, whereas HA and CO_3_^2−^ slightly inhibited the reaction, especially at higher concentrations. This inhibition may result from competitive consumption of reactive species or occupation of active sites by natural organic matter and inorganic carbon species. The pH-dependent experiments showed that E2 degradation was more favorable under weakly acidic to neutral conditions, particularly at pH 5–7, while acidic and alkaline conditions suppressed the reaction to varying degrees (Figure 5b). This behavior may be related to changes in PAA speciation, surface charge properties, and Co redox cycling. The stability and reusability of Co@C_in_ were evaluated over five consecutive cycles (Figure 5c). Although a slight decrease in degradation rate was observed after repeated use, Co@C_in_ maintained considerable catalytic activity throughout the cyclic tests, indicating satisfactory reusability. Overall, these results demonstrate that the nanoconfined Co@C_in_ catalyst can efficiently activate PAA for E2 degradation over a broad range of operating conditions, with good resistance to interference from common water constituents and satisfactory reusability.

In addition to catalytic reusability, Co leaching was also evaluated to assess the stability of the confined Co species and the potential risk of secondary metal release. After 30 min of reaction under the standard conditions, the dissolved Co concentration in the Co@C_in_/PAA system was only 4.8 μg L^−1^, whereas a substantially higher Co concentration of 38.6 μg L^−1^ was detected in the Co@C_out_/PAA system. Considering the actual Co loadings of the two catalysts, the corresponding Co-leaching fractions were approximately 0.0099% and 0.0811%, respectively. Thus, the amount of Co released from Co@C_in_ was approximately one-eighth of that from Co@C_out_.

The substantially suppressed Co leaching from Co@C_in_ indicates that confinement of the Co species within the CNT nanochannels effectively stabilizes the Co-containing domains and limits their direct exposure to the bulk solution. Combined with the maintained catalytic performance over five consecutive cycles, the low Co release supports the satisfactory operational stability of Co@C_in_ and further highlights the beneficial role of nanoconfinement in suppressing secondary Co release.

### 2.3. Mechanistic Insights and Possible Transformation Routes of E2 in the Co@C_in_/PAA System

**Figure 6:**
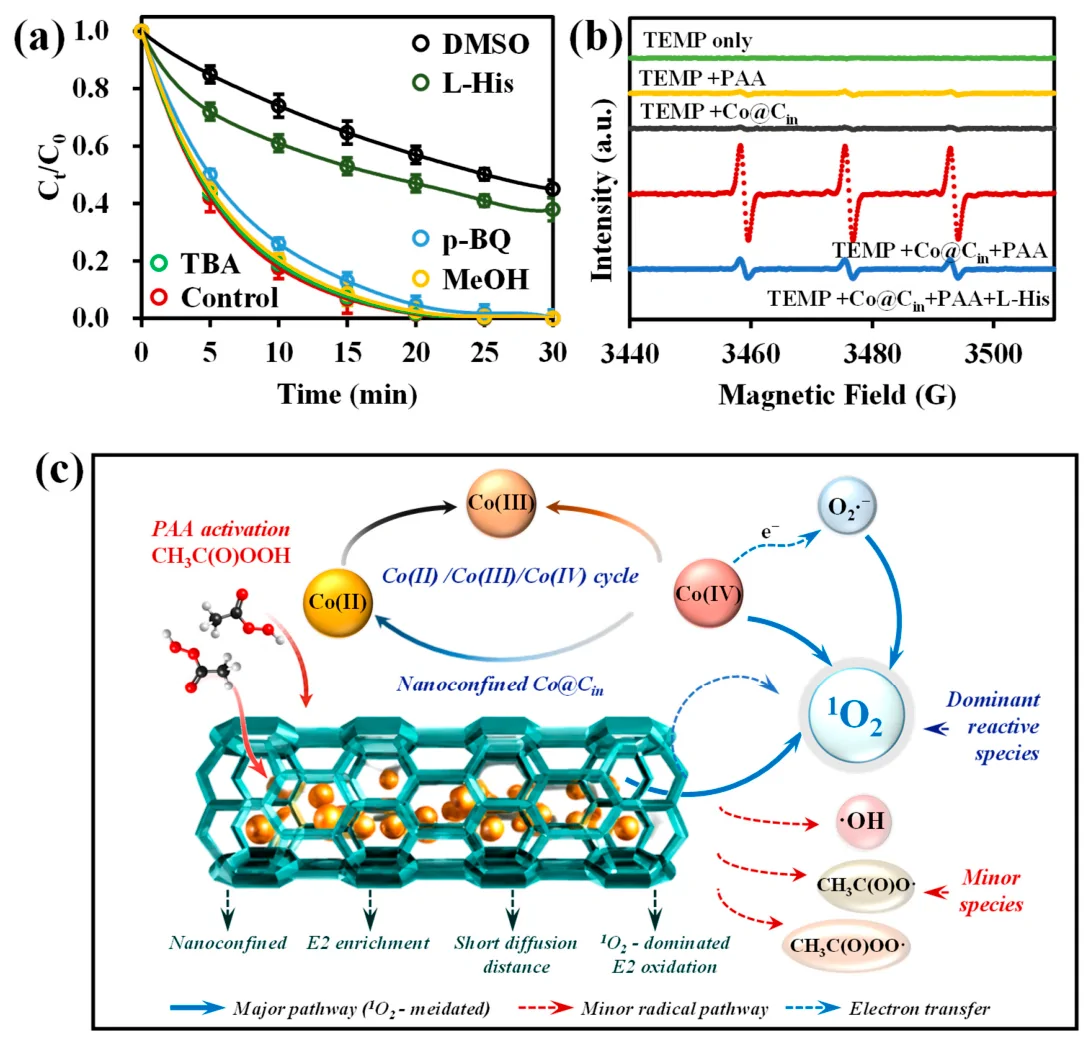
Mechanistic investigation of E2 degradation in the Co@C_in_/PAA system: (**a**) effect of different scavengers on E2 degradation; (**b**) TEMP-EPR spectra for ^1^O_2_ detection; and (**c**) proposed catalytic mechanism for E2 degradation. The Co(IV)-oxo species and Co(II)/Co(III)/Co(IV) pathway shown in panel (**c**) represent a literature-supported mechanistic hypothesis and were not directly analyzed in the present study.

To identify the major reactive species involved in the Co@C_in_/PAA system, scavenging experiments were performed using different selective quenchers (Figure 6a). The complete E2/Co@C_in_/PAA system without the addition of any scavenger was used as the control. Compared with the control, the addition of TBA and MeOH caused only limited inhibition of E2 degradation, while p-BQ exhibited a negligible effect. In contrast, L-histidine markedly suppressed E2 degradation, indicating an important contribution from ^1^O_2_. DMSO also caused a certain degree of inhibition, suggesting that high-valent Co species may participate in the reaction process. The involvement of ^1^O_2_ was further examined by TEMP-EPR spectroscopy. As displayed in Figure 6b, a pronounced characteristic three-line TEMPO signal was observed in the complete Co@C_in_/PAA system, whereas the corresponding control systems exhibited negligible or substantially weaker signals. Together with the L-His scavenging results, these observations support an important contribution of ^1^O_2_. Combined with the quenching results, these findings indicate that E2 degradation over Co@C_in_/PAA proceeded predominantly through a nonradical^1^O_2_-mediated pathway, whereas ·OH, O_2_•^−^, and organic acyl/peroxy radicals served only as minor reactive species [46].

Based on the above results, a possible catalytic mechanism is proposed (Figure 6c). PAA is first adsorbed and activated by the mixed-valence Co sites confined within the CNT nanochannels. Based on the mixed Co(II)/Co(III) states identified by XPS and previously reported Co/PAA chemistry, Co(II)/Co(III) redox transformation is proposed to participate in PAA activation. The involvement of transient high-valent Co species, such as Co(IV)-oxo, is considered a possible pathway based on the partial inhibition observed with DMSO and previous literature, but was not directly demonstrated in the present study. Accordingly, the Co(II)/Co(III)/Co(IV) pathway illustrated in Figure 6c should be regarded as a literature-supported mechanistic hypothesis rather than a directly verified catalytic cycle. Meanwhile, the hollow CNT channels provide a nanoconfined reaction microenvironment that can enrich E2 and PAA, shorten the diffusion distance between reactants and active sites, and suppress the random release of highly reactive radical species into the bulk solution. Consequently, Co@C_in_ facilitates efficient E2 degradation predominantly through a ^1^O_2_-mediated nonradical oxidation pathway, while radical-mediated reactions make only minor contributions [47,48]. Based on this experimentally supported reactive-species mechanism and previously reported E2 oxidation chemistry, possible molecular transformation routes of E2 are proposed.

Although the transformation intermediates of E2 were not directly identified in the present study, the experimentally observed dominant contribution of ^1^O_2_ provides a basis for discussing its possible molecular transformation behavior. E2 contains an electron-rich phenolic A-ring and a secondary hydroxyl group at the C17 position, both of which have been reported to be susceptible sites during oxidative transformation [20]. Previous studies have shown that E2 may undergo oxidation of the C17 hydroxyl group to form estrone (E1), as well as hydroxylation of the phenolic A-ring to generate catechol-type products such as 2-hydroxyestradiol and 4-hydroxyestradiol [49,50]. Further oxidation of these hydroxylated products may lead to quinone-like intermediates, followed by oxidative cleavage of the aromatic A-ring and formation of ring-opened oxygenated products [51]. Continued oxidation may ultimately generate lower-molecular-weight oxygenated compounds, including dicarboxylic acids [49]. Therefore, based on the dominant role of ^1^O_2_ observed in the Co@C_in_/PAA system and previously reported E2 oxidation chemistry, phenolic A-ring oxidation and C17 hydroxyl oxidation are considered possible transformation routes for E2 [20,49,50,51]. It should be emphasized that these transformation routes are literature-supported hypotheses rather than experimentally confirmed pathways in the present system, and direct identification by LC–MS/MS or high-resolution mass spectrometry will be required for further verification. It should also be noted that the present HPLC analysis confirms the disappearance of the parent E2 molecule but does not directly demonstrate elimination of the estrogenic activity of its transformation products. Evaluation of residual estrogenicity will therefore be necessary in future work [16,21].

## 3. Materials and Methods

### 3.1. Materials

E2 with a purity > 99%, CNTs and HPLC-grade methanol, ethanol, and acetonitrile were purchased from Aladdin (Shanghai, China). Co(NO_3_)_2_·6H_2_O, sodium thiosulfate (Na_2_S_2_O_3_), sodium hydrogen carbonate (NaHCO_3_), potassium nitrate (KNO_3_), sodium chloride (NaCl), sodium hydroxide (NaOH), and hydrochloric acid (HCl), all analytical grade, were purchased from Merck (Shanghai, China). Commercial PAA solution (~15% PAA, 24% H_2_O_2_, and 16% acetic acid), hydrogen peroxide (H_2_O_2_, 30%) and nitric acid (HNO_3_) were obtained from Nanjing Chemical Reagent Co., Ltd. (Nanjing, China). Analytical-grade tert-butyl alcohol (TBA), dimethyl sulfoxide (DMSO), p-benzoquinone (p-BQ), L-histidine (L-His), and 2,2,6,6-tetramethylpiperidine (TEMP) were also purchased from Aladdin (Shanghai, China). Milli-Q water was produced using an XUC-20L ultrapure water purification system (Xiniu, Shanghai, China).

### 3.2. Preparation of Co@C_in_ Catalyst

**Figure 7:**
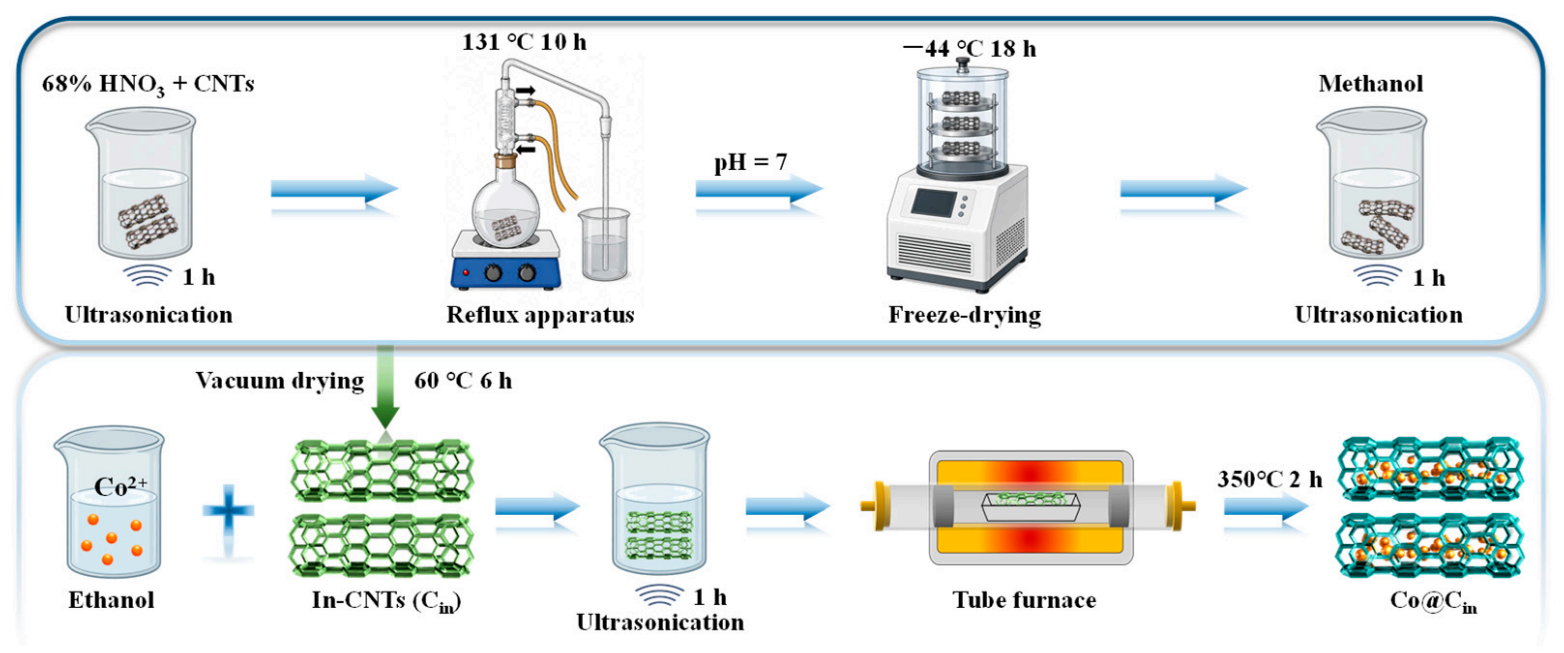
Diagram of Co@C_in_ preparation.

The nanoconfined Co-based CNTs, denoted as Co@C_in_, were prepared through a broken-carbon nanotube-assisted impregnation and confined pyrolysis strategy. Briefly, 0.5 g of pristine CNTs was dispersed in 100 mL of HNO_3_ solution and stirred at room temperature for 1 h. The suspension was then refluxed at 131 °C for 10 h under continuous stirring. After cooling to room temperature, the product was filtered through a 0.22 μm membrane, washed repeatedly with ultrapure water to neutral pH, and freeze-dried to obtain broken CNTs. Subsequently, 0.4 g of Co(NO_3_)_2_·6H_2_O and 0.2 g of b-CNTs were separately dispersed in methanol by ultrasonication, mixed, stirred for 1 h, and further ultrasonicated for 2.5 h to promote the diffusion of Co precursors into the inner cavity of b-CNTs. The solid was collected by filtration, washed with ethanol and water, and dried at 60 °C. Finally, the obtained precursor was calcined under N_2_ at 350 °C for 2 h under a heating rate of 1 °C min^−1^. After washing and vacuum drying, Co@C_in_ was obtained (Figure 7). For comparison, the non-confined catalyst Co@C_out_ was prepared using defective CNTs without opening the tube ends. The Co precursor was loaded mainly on the outer surface of CNTs under similar impregnation and calcination conditions [43].

### 3.3. Characterization

Scanning electron microscopy (SEM, Sigma 360, ZEISS, Oberkochen, Germany) coupled with energy-dispersive X-ray spectroscopy (EDS) was used to observe the surface morphology and elemental distribution of C, O, and Co. Transmission electron microscopy (TEM) and high-resolution TEM were performed on a Tecnai F20 microscope (FEI, Morristown, NJ, USA) to examine the dispersion and spatial location of Co species inside or outside the CNT nanochannels. Before TEM analysis, the samples were ultrasonically dispersed in anhydrous ethanol and dropped onto carbon-coated copper grids. The crystalline structures were analyzed by X-ray diffraction (XRD, Ultima IV, Rigaku, Tokyo, Japan) using Cu Kα radiation over a 2θ range of 10–80° at 40 kV and 40 mA. Raman spectra were recorded using an HR Evolution Raman spectrometer (Horiba Scientific, Kyoto, Japan) with a 532 nm laser in the range of 100–3000 cm^−1^. The I_D_/I_G_ ratio was calculated from the measured peak intensities of the D and G bands without additional peak deconvolution. X-ray photoelectron spectroscopy (XPS, ESCALAB 250Xi, Thermo Scientific, Waltham, MA, USA) was used to analyze the surface elemental composition and Co valence states. The C 1s and Co 2p spectra were fitted using Avantage software (version 6.6.0, Thermo Fisher Scientific, Waltham, MA, USA). The actual Co contents of Co@C_in_ and Co@C_out_ were determined by inductively coupled plasma optical emission spectrometry (ICP-OES, Agilent 5110, Agilent Technologies, Santa Clara, CA, USA). Approximately 20.0 mg of each catalyst was placed in a PTFE microwave digestion vessel, followed by the addition of 9 mL of concentrated HNO_3_ and 1 mL of 30% H_2_O_2_. The samples were subjected to microwave-assisted digestion at 180 °C for 15 min. After cooling to room temperature, the digests were quantitatively transferred to 50 mL volumetric flasks and diluted to the appropriate volume with ultrapure water. The resulting solutions were further diluted when necessary to fall within the ICP-OES calibration range. The Co loading was calculated from the measured Co concentration and the initial catalyst mass. All measurements were performed in triplicate.

### 3.4. Experimental Procedures

The catalytic degradation of E2 was carried out in 250 mL glass reactors with a working volume of 100 mL. Unless otherwise specified, the typical reaction system contained 2 mg/L E2, 0.20 g/L Co@C_in_, and 500 μM PAA at an initial pH of 7.0. The experiments were performed at 25 ± 2 °C under magnetic stirring or shaking at 200 rpm. Prior to oxidation, the catalyst was added to the E2 solution and equilibrated in the dark for 30 min to minimize the influence of adsorption. The reaction was initiated by adding PAA, and this moment was defined as t = 0. Control experiments, including PAA alone, CNTs, Co@C_in_ alone, Co@C_out_/PAA, and Co@C_in_/PAA, were conducted to distinguish adsorption, direct oxidation, and catalytic activation contributions. The effects of key operational parameters were evaluated by varying one factor while maintaining the others unchanged. The tested ranges included E2 concentrations of 1–5 mg/L, catalyst dosages of 0.05–0.25 g/L, and PAA concentrations of 100–1000 μM. At predetermined intervals, 1.0 mL aliquots were withdrawn, immediately quenched with excess Na_2_S_2_O_3_, and filtered through a 0.22 μm membrane before HPLC analysis. All experiments were conducted at least in duplicate, and the data are presented as mean values with standard deviations. E2 was quantified using a high-performance liquid chromatography instrument (HPLC, Shimadzu SPD-20A UV detection system, Shimadzu, Kyoto, Japan) equipped with a UV detector and a C18 reversed-phase column (250 mm × 4.6 mm, 5 μm). Methanol/water (70/30, *v*/*v*) was used as the mobile phase under isocratic elution at a flow rate of 1.0 mL/min. The detection wavelength was 280 nm, and the injection volume was 10 μL. E2 concentrations were determined using an external calibration method. Catalyst reusability was assessed over five consecutive cycles. After each run, Co@C_in_/PAA was recovered, washed with ultrapure water and ethanol, dried under vacuum at 60 °C, and reused under identical conditions.

Quenching experiments were performed by individually adding TBA (100 mM), MeOH (100 mM), DMSO (100 mM), p-BQ (1 mM), and L-His (10 mM) to the standard Co@C_in_/PAA/E2 system under otherwise identical conditions. TBA and MeOH were used to evaluate radical-mediated pathways, whereas DMSO, p-BQ, and L-His were employed to probe the possible contributions of high-valent Co species, O_2_•^−^, and ^1^O_2_, respectively. TEMP-EPR spectroscopy was employed to further examine the involvement of ^1^O_2_ (EPR, MS-5000 Spectrometer Bruker, Germany) using TEMP (20 mM) as the spin probe. EPR measurements were performed for TEMP alone, TEMP/PAA, TEMP/Co@C_in_, and TEMP/Co@C_in_/PAA under otherwise identical conditions. An additional experiment was conducted by adding L-His (10 mM) to the complete TEMP/Co@C_in_/PAA system to evaluate its influence on the characteristic three-line TEMPO signal [43].

The degradation kinetics of E2 were evaluated using a pseudo-first-order kinetic model:

$$\ln\left(\frac{C_{0}}{C_{t}}\right)=k_{obs}t$$

where *C*_0_ and *C_t_* represent the E2 concentrations at *t* = 0 and reaction time *t*, respectively, and *k_obs_* is the apparent pseudo-first-order rate constant (min^−1^). The corresponding half-life was calculated according to:

$$t_{1/2}=\frac{ln2}{k_{obs}}$$

Linear regression of ln(*C*_0_/*C_t_*) versus reaction time was used to determine k_obs_ and R^2^. To compare Co@C_in_/PAA and Co@C_out_/PAA, kinetic fitting was performed using the data within 0–20 min.

To evaluate Co leaching, reaction suspensions were collected after 30 min under the standard reaction conditions. The solid catalysts were removed by centrifugation followed by membrane filtration, and the resulting solutions were acidified with ultrapure HNO_3_ prior to analysis. The dissolved Co concentrations were quantified by ICP-MS (Agilent 7900, Agilent Technologies, Santa Clara, CA, USA). The same procedure was applied to both Co@C_in_/PAA and Co@C_out_/PAA systems. The Co-leaching fraction was calculated as the amount of dissolved Co relative to the total Co initially introduced with the catalyst.

## 4. Conclusions

In this study, a nanoconfined Co-based catalyst, Co@C_in_, was applied for PAA activation for the degradation of E2. Compared with PAA alone, CNTs, Co@C_in_ alone, and the externally loaded Co@C_out_/PAA system, Co@C_in_/PAA exhibited substantially enhanced E2 degradation performance, demonstrating the performance advantage of the internally confined configuration for E2 oxidation.

Operational studies demonstrated that E2 degradation was influenced by pollutant concentration, catalyst dosage, PAA concentration, and solution pH, with efficient removal achieved under near-neutral conditions. Common water constituents such as Cl^−^ and NO_3_^−^ showed limited interference, whereas humic acid and CO_3_^2−^ caused slight inhibition. Co@C_in_ also maintained considerable activity over five consecutive cycles. Mechanistic investigations indicated that ^1^O_2_ played an important role in the Co@C_in_/PAA system, whereas radical pathways contributed to a lesser extent. These results extend our previous understanding of nanoconfinement-regulated PAA activation by demonstrating its applicability to the systematic oxidation of the highly bioactive steroid estrogen E2 and by directly comparing the performance of internally confined and externally loaded Co species. Overall, this study extends nanoconfinement-regulated PAA oxidation to the treatment of 17β-estradiol and demonstrates the potential of internally confined Co species for efficient steroid estrogen removal.

## Data Availability

The original contributions presented in this study are included in the article. Further inquiries can be directed to the corresponding author.
